# Homo- and Heterosubtypic Low Pathogenic Avian Influenza Exposure on H5N1 Highly Pathogenic Avian Influenza Virus Infection in Wood Ducks (*Aix sponsa*)

**DOI:** 10.1371/journal.pone.0015987

**Published:** 2011-01-06

**Authors:** Taiana P. Costa, Justin D. Brown, Elizabeth W. Howerth, David E. Stallknecht, David E. Swayne

**Affiliations:** 1 Department of Pathology, College of Veterinary Medicine, The University of Georgia, Athens, Georgia, United States of America; 2 Southeastern Cooperative Wildlife Disease Study, Department of Population Health, College of Veterinary Medicine, The University of Georgia, Athens, Georgia, United States of America; 3 Southeast Poultry Research Laboratory, United States Department of Agriculture, Agricultural Research Service, Athens, Georgia, United States of America; Centers for Disease Control and Prevention, United States of America

## Abstract

Wild birds in the Orders Anseriformes and Charadriiformes are the natural reservoirs for avian influenza (AI) viruses. Although they are often infected with multiple AI viruses, the significance and extent of acquired immunity in these populations is not understood. Pre-existing immunity to AI virus has been shown to modulate the outcome of a highly pathogenic avian influenza (HPAI) virus infection in multiple domestic avian species, but few studies have addressed this effect in wild birds. In this study, the effect of pre-exposure to homosubtypic (homologous hemagglutinin) and heterosubtypic (heterologous hemagglutinin) low pathogenic avian influenza (LPAI) viruses on the outcome of a H5N1 HPAI virus infection in wood ducks (*Aix sponsa*) was evaluated. Pre-exposure of wood ducks to different LPAI viruses did not prevent infection with H5N1 HPAI virus, but did increase survival associated with H5N1 HPAI virus infection. The magnitude of this effect on the outcome of the H5N1 HPAI virus infection varied between different LPAI viruses, and was associated both with efficiency of LPAI viral replication in wood ducks and the development of a detectable humoral immune response. These observations suggest that in naturally occurring outbreaks of H5N1 HPAI, birds with pre-existing immunity to homologous hemagglutinin or neuraminidase subtypes of AI virus may either survive H5N1 HPAI virus infection or live longer than naïve birds and, consequently, could pose a greater risk for contributing to viral transmission and dissemination. The mechanisms responsible for this protection and/or the duration of this immunity remain unknown. The results of this study are important for surveillance efforts and help clarify epidemiological data from outbreaks of H5N1 HPAI virus in wild bird populations.

## Introduction

Wild birds, especially those in the Orders Anseriformes and Charadriiformes, are the natural reservoirs for all known hemagglutinin (HA) (H1–H16) and neuraminidase (NA) (N1–N9) subtypes of avian influenza (AI) viruses [1], [2], [3], [4], [5]. It has been further suggested that wild birds might be involved in the geographic spread, transmission to humans, and maintenance of H5N1 highly pathogenic avian influenza (HPAI) virus in Eurasia and Africa [6], [7], [8].

Concurrent or successive infections with different low pathogenic avian influenza (LPAI) virus subtypes have been reported in both wild bird populations and poultry [9], [10], [11], [12]. Existing experimental data suggest that AI virus infection in waterfowl is associated with a variable protective immune response. In Pekin ducks (*Anas platyrhynchos domesticus*) primary inoculation with a LPAI virus induced partial protection against repeated inoculation with the same virus [13]. Additionally, pre-exposure to LPAI viruses has been shown to induce partial protection against H5N1 HPAI virus infection in Canada geese (*Branta canadensis*) [14], [15], mute swans (*Cygnus olor*) [16], mallards (*Anas platyrhynchos*) [17], and chickens [18], [19], [20] as evidenced by reduced morbidity, mortality, susceptibility, and/or viral shedding. Such protective immunity may be one possible explanation for unexpected field observations during natural outbreaks of H5N1 HPAI virus, including lower than anticipated morbidity in poultry in Hong Kong [21] and wild birds in Germany despite the presence of several hundred thousand susceptible birds in the affected area [22]. The mechanisms of this protective effect, however, are still poorly defined.

To better understand the epidemiology of H5N1 HPAI virus infections in wild bird populations, we experimentally investigated the effects that pre-exposure with homosubtypic (homologous HA) (H5N1 or H5N2) or heterosubtypic (heterologous HA) (H1N1) LPAI viruses had on the outcome of a Eurasian H5N1 HPAI virus challenge in wood ducks (*Aix sponsa*).

## Materials and Methods

### Ethics Statement

General animal care was provided under an Animal Use Protocol (A2007-10058-0) approved by the Institutional Animal Care and Use Committee at The University of Georgia and the Southeast Poultry Research Laboratory (SEPRL), United States Department of Agriculture, Agricultural Research Service (Athens, Georgia, USA).

### Animals

Twenty-five 4-month-old wood ducks were purchased from a waterfowl breeder. Both males and females were included in approximately equal numbers. Food and water were provided *ad libitum*. The wood duck was selected as the animal model due to the availability of data on the susceptibility, viral shedding patterns, and pathobiology associated with the H5N1 HPAI virus used in this study [23], [24].

### Viruses

Three North American and one Eurasian wild bird-origin LPAI viruses were selected for the pre-exposure portion of this study. The LPAI virus strains included: A/mallard/MN/355779/00 (H5N2) and A/blue-winged teal/LA/B228/86 (H1N1) (Southeastern Cooperative Wildlife Disease Study [SCWDS], Athens, Georgia, USA), A/mute swan/MI/451072-2/06 (H5N1) (SEPRL, Athens, Georgia, USA), and A/mallard/Netherlands/2/05 (H5N2) (Ron Fouchier, Erasmus University, Rotterdam, The Netherlands). The HPAI isolate A/whooper swan/Mongolia/244/05 (H5N1) (Mongolia/05) (SEPRL, Athens, Georgia, USA) was used as challenge virus. The Mongolia/05 virus is a clade 2.2 virus and is genetically representative of H5N1 HPAI viruses that have been reported in wild birds in Asia, Europe, and Africa [24]. The virus was originally isolated from a dead whooper swan during a large die-off of waterfowl, and it was chosen for use in this study because of its established lethality in wild waterfowl, particularly wood ducks [23], [24], [25].

Individual stocks of the LPAI and the HPAI viruses used in this study were produced by second passage in 9-day-old specific pathogen free (SPF) embryonating chicken eggs (ECE). Viral stocks were titrated in SPF ECE and median embryo infectious dose (EID_50_) titers were calculated using previously described methods [26]. Viral stocks were diluted in sterile brain-heart-infusion (BHI) medium to yield a final titer of 10^6^ EID_50_ per 0.1 mL for LPAI viruses and 10^4^ per 0.1 mL for the H5N1 HPAI virus (single bird inoculum).

### Experimental Design

Wood ducks were evenly divided into five groups. Each group was housed separately in biocontainment isolation units ventilated under negative pressure with a high efficiency particulate air filters on intake and exhaust. After four days of acclimation, four of the groups were inoculated via choanal cleft into the middle nasal cavity with 0.1 mL of one of the four LPAI viruses and designated accordingly: A/mallard/Netherlands/2/05 (H5N2) group; A/blue-winged teal/LA/B228/86 (H1N1) group; A/mute swan/MI/451072-2/06 (H5N1) group; and A/mallard/MN/355779/00 (H5N2) group. One group was not experimentally exposed to a LPAI virus and served as a negative control (naïve group). Blood was collected from all birds prior to LPAI virus inoculation and at 21 days post-LPAI virus exposure (LPAIV-dpe) to test for the presence of antibodies to AI virus. In addition, cloacal and oropharyngeal (OP) swabs were collected from each bird for virus isolation prior to the study and at 2, 4, 7, and 21 LPAIV-dpe. After LPAI virus inoculation, birds were observed daily for behavior changes and/or the development of any clinical signs. At 21 LPAIV-dpe, the four LPAI virus exposed groups and the naïve group were inoculated via choanal cleft with 0.1 mL of the Mongolia/05 virus. Cloacal and OP swabs were collected from all the birds at 0, 1, 2, 4, 8, and 10 days post-HPAI virus challenge (HPAIV-dpc), and from any birds that were found dead or were euthanized due the severity of their clinical condition. Blood samples were collected at 10 HPAIV-dpc to test for the presence of antibodies to AI virus. Birds were evaluated daily as described above. Wood ducks exhibiting severe clinical signs (paresis or paralysis and/or inability to eat or drink) were euthanized with intravenous administration of sodium pentobarbital (100 mg/kg), and a full necropsy was performed. The experiment was terminated at 10 HPAIV-dpc at which time all the remaining birds were euthanized, as described above, and full necropsies were performed. All experimental infections were performed in a Biosafety Level-3-Enhanced facility at the SEPRL.

### Serology

All blood samples were collected from the right jugular vein. Serum samples were stored at −20°C until tested for the presence of influenza A antibodies using two serologic assays. Samples were tested for antibodies directed against the conserved internal nucleoprotein of influenza A viruses using a blocking enzyme-linked immunosorbent assay (bELISA) (FlockChek Avian Influenza MultiS-Screen Antibody Test Kit, IDEXX Laboratories, Westbrook, Maine, USA) following the manufacturer's instructions. Additionally, all samples were further analyzed for presence of antibodies directed against specific HA subtypes using the hemagglutination inhibition (HI) test following procedures previously described [27]. The LPAI viruses used for the pre-exposure portion of the study and the Mongolia/05 challenge virus served as antigens for the HI test. Each treatment group was tested against the LPAI virus used for pre-exposure and all the groups were tested against the Mongolia/05. A 0.5% suspension of chicken erythrocytes in phosphate-buffered saline was used for the HI tests. Serum samples were pre-treated with chicken red blood cells prior to the HI test in order to neutralize any naturally occurring serum hemagglutinins. HI titers≥8 were considered positive.

### Virus Isolation

Cloacal and OP swabs were collected in sterile BHI medium with antimicrobial drugs (100 µg/mL gentamicin, 100 units/mL penicillin, and 5 µg/mL amphotericin B). Samples were stored at −70°C until testing was performed. Isolation of virus was performed by inoculating the allantoic sac of four 9-day-old SPF ECE with the clarified BHI media containing the swab (0.25 mL per egg). Allantoic fluid was harvested from all inoculated SPF ECE after 4 days of incubation at 37°C and tested for the presence of hemagglutinating activity [28]. Any bird was considered positive in which one or more inoculated SPF ECE tested positive for the presence of virus on cloacal swabs at any time point after inoculation and/or on OP swabs at the second day post inoculation or later (to minimize the potential for identifying residual inoculum on oropharynx).

### Histopathology and Immunohistochemistry

Samples of cerebrum, cerebellum, heart, lung, trachea, liver, spleen, esophagus, proventriculus, ventriculus, small intestine, large intestine, pancreas, adrenal, ovaries/testis, kidney, cloacal bursa, pectoral muscle, femur, nasal turbinates and sinus were collected from all the animals and fixed in 10% buffered formalin for histopathology and immunohistochemistry (IHC). After fixation, the tissues were processed and embedded in paraffin, and 5µm sections were stained with hematoxylin and eosin using standard histopathology protocols. Femur and nasal turbinates were decalcified with Kristensen's decalcifying solution before being processed. Duplicate sections of all tissues were immunohistochemically stained by using a mouse-derived monoclonal antibody (P13C11) specific for type A influenza virus nucleoprotein antigen as the primary antibody (SEPRL, Athens, Georgia, USA). Procedures used to perform the immunohistochemical testing have been previously described [29]. Fast red was used as the substrate chromogen, and slides were counterstained with hematoxylin. Demonstration of viral antigen was based on chromogen deposition in the nucleus, with or without chromogen deposition in the cytoplasm.

### Statistical Analysis

Fisher Exact Test with a level of significance (α) of 0.05 was used to evaluate the association between pre-exposure to each of the LPAI viruses and the survival following Mongolia/05 virus challenge. Chi-square (χ^2^) distribution with α of 0.05 was employed to analyze the relation between survival following Mongolia/05 virus challenge and the serological status upon arrival.

## Results

### Pre-trial Status

Prior to the start of the study (0 LPAIV-dpe), 12 of the 25 wood ducks tested positive for influenza A virus nucleoprotein antibodies with the bELISA (Table 1). These 12 birds were distributed among the naïve and the four LPAI virus exposure groups. All 25 serum samples collected at 0 LPAIV-dpe were negative on HI tests, indicating the absence of pre-existing antibodies directed against the HA of the pre-exposure LPAI virus or the Mongolia/05 virus. Virus was not isolated from any cloacal or OP swabs collected at 0 LPAIV-dpe, indicating that no birds were actively infected at the start of the study.

**Table 1:** Serological status before and after the experimental low pathogenicity avian influenza (LPAI) virus exposure, virus isolation data, and mortality of wood ducks (*Aix sponsa*) experimentally inoculated^a^ with different subtypes of LPAI viruses and subsequently challenged with the highly pathogenic avian influenza (HPAI) virus A/whooper swan/Mongolia/244/05 (H5N1)^b^.

		LPAI virus Pre-exposure		HPAI virus Challenge			
	Serological status upon arrival	Virus Isolation	Serology	Virus Isolation	Mortality		
GROUPS^c^	bELISA result (n)^e^^,^ f	Prevalence^g^, no. positive/total	HI^h^ no. positive/total	Prevalence, no. positive/total	no. deaths/total (%)	% (n = 5)	MDT (d) (n = 5)
Naïve^d^	+ (1)	NA	NA	1/1	1/1 (100)	100	5
	− (4)	NA	NA	4/4	4/4 (100)		
A/mallard/Netherlands/2/05 (H5N2)	+ (2)	0/2	0/2	2/2	2/2 (100)	80	6
	− (3)	0/3	0/3	3/3	2/3 (66)		
A/blue-winged teal/LA/B228/86 (H1N1)	+ (2)	2/2	2/2	2/2	1/2 (50)	60	6
	− (3)	2/3	1/3	3/3	2/3 (66)		
A/mute swan/MI/451072-2/06 (H5N1)	+ (3)	0/3	0/3	3/3	1/3 (33)	20	8
	− (2)	2/2	0/2	2/2	0/2 (0)		
A/mallard/MN/355779/00 (H5N2)	+ (4)	4/4	3/4	4/4	0/4 (0)	0	NA
	− (1)	1/1	1/1	1/1	0/1 (0)		

a Birds were pre-exposed via choanal cleft with a dose of 10^6^EID_50_.

b Birds were challenged via choanal cleft with a dose of 10^4^EID_50_ 21 days after experimental pre-exposure to LPAI viruses.

c Groups of wood ducks (n = 5) experimentally pre-exposed to different LPAI viruses.

d The naïve group was not experimentally pre-exposed to LPAI virus.

e Abbreviations: bELISA = blocking ELISA; HI = hemagglutination inhibition; MDT = mean death time (days); + = positive; − = negative; NA = non applicable.

f bELISA result (number of birds). Twelve out of 25 birds had avian influenza nucleoprotein antibodies at the beginning of the trial, before experimental exposure to a LPAI virus.

g Number of birds that had at least one cloacal and/or oropharyngeal swab that tested positive on virus isolation after avian influenza virus exposure/total number of birds.

h HI using antigen against homosubtypic LPAI virus, either H5N2, H1N1, or H5N1. Serum samples were colleted 21 days after LPAI virus pre-exposure.

### LPAI Pre-exposure

All wood ducks in each of the five groups remained clinically and behaviorally normal, and had an average weight gain of 8.9% (2.0–15.3%) during the 21 days following the LPAI virus pre-exposure (data not shown).

Detection of viral shedding (Table 1) and seroconversion, based on the HI test (Tables 1 and 2), varied between LPAI-exposure groups. The A/mallard/Netherlands/2/05 (H5N2) virus did not replicate in any of the inoculated wood ducks, as demonstrated by lack of viral shedding and seroconversion (by both the bELISA and HI tests). The A/mute swan/MI/451072-2/06 (H5N1) virus was recovered from two of the five inoculated wood ducks; in both ducks virus was isolated from OP swabs collected at 2 LPAIV-dpe. Although both of these ducks were seronegative at 21 LPAIV-dpe on the H5 HI test (Table 2), antibodies were detected in one duck with the bELISA.

**Table 2:** Serological status, as determined by hemagglutination inhibition, of wood ducks (*Aix sponsa*) 21 days after experimental pre-exposure to low pathogenicity avian influenza (LPAI) viruses^a^, and 10 days after challenge with the highly pathogenic avian influenza (HPAI) virus A/whooper swan/Mongolia/244/05 (H5N1)^b^.

	Hemagglutination Inhibition Titer^c^		
	Homosubtypic LPAI virus antigen^d^		A/whooper swan/244/05 antigen^e^
Sera from exposed birds by group, bird ID	21 LPAIV-dpe^f^	10 HPAIV-dpc	10 HPAIV-dpc
A/blue-winged teal/LA/B228/86 (H1N1)			
11	<8	†	†
12	8	8	<8
13	8	†	†
14	16	16	<8
15	<8	†	†
A/mute swan/MI/451072-2/06 (H5N1)			
6	<8	128	16
7	<8	†	†
8	<8	256	128
9	<8	128	32
10	<8	256	512
A/mallard/MN/355779/00 (H5N2)			
16	8	128	8
17	<8	256	<8
18	16	64	<8
19	16	1024	32
20	8	32	<8

a Birds were inoculated via choanal cleft with a dose of 10^6^EID_50_ of different LPAI viruses. Serologic data of the naïve group were omitted due the 100% mortality observed in this group, and data of the A/mallard/Netherlands/2/05 (H5N2) group were omitted due to lack of seroconversion.

b Birds were challenged via choanal cleft with a dose of 10^4^EID_50_ of A/whooper swan/Mongolia/244/05 (H5N1), 21 days after experimental pre-exposure to different subtypes of LPAI viruses.

c Samples with HI titer≥8 were considered positive.

d HI using antigen against homosubtypic LPAI virus, either H1N1, H5N1, or H5N2.

e HI using antigen against A/whooper swan/Mongolia/244/05 (H5N1).

f Abbreviations: LPAIV-dpe = days after LPAI pre-exposure; HPAIV-dpc = days after H5N1 HPAI challenge; † = succumbed to HPAI H5N1 infection.

Viral shedding was detected in four of five wood ducks inoculated with the A/blue-winged teal/LA/B228/86 (H1N1) virus. Virus was reisolated from wood ducks in this group until 4 to 7 LPAIV-dpe (mean 4.75 days). Three out of the four ducks that excreted the A/blue-winged teal/LA/B228/86 (H1N1) virus seroconverted, as detected by H1 specific HI test; two of these three wood ducks also had detectable antibodies on bELISA at 21 LPAIV-dpe. The A/mallard/MN/355779/00 (H5N2) virus replicated efficiently in all the five inoculated wood ducks independently of the serological status at 0 LPAIV-dpe. Viral shedding in this group was detected until 2 to 4 LPAIV-dpe (mean 2.4 days). Seroconversion was detected in four of five wood ducks in this group with both the bELISA and H5 HI test.

### A/whooper swan/Mongolia/244/05 (H5N1) Challenge

#### Serological Status

Serological status for each of the exposure groups, as determined by the HI test at 21 LPAIV-dpe (against the homosubtypic LPAI viruses used for pre-exposure) and at 10 HPAIV-dpc (against Mongolia/05 virus), is summarized in Table 2. Serological data for the naïve group were omitted from Table 2 because all the wood ducks in this group died after Mongolia/05 virus challenge. Data from the A/mallard/Netherlands/2/05 (H5N2) group were also omitted from Table 2 because none of these birds seroconverted after the LPAI virus pre-exposure, and four of five died after Mongolia/05 virus challenge. The only wood duck in this group that survived the Mongolia/05 virus challenge had an HI titer of 16 at 10 HPAIV-dpc.

Following the Mongolia/05 virus challenge (at 10 HPAIV-dpc), all of the surviving wood ducks in the A/mute swan/MI/451072-2/06 (H5N1) and A/mallard/MN/355779/00 (H5N2) groups had an increase in the HI titer against the LPAI virus used for pre-exposure. Three wood ducks that succumbed to Mongolia/05 virus infection (two birds in the A/blue-winged teal/LA/B228/86 (H1N1) group and one bird in the A/mute swan/MI/451072-2/06 (H5N1) group) had negative HI titers (<8) against the LPAI virus used for pre-exposure at 21 LPAIV-dpe (Table 2).

#### Morbidity and Mortality

Wood ducks that succumbed to the Mongolia/05 virus challenge (n = 13) progressively lost weight, while the ducks that survived (n = 12) experienced an initial weight loss during the first days after Mongolia/05 virus challenge, followed by a progressive weight gain throughout the remainder of the trial (data not shown). All wood ducks that succumbed to infection either exhibited premonitory neurologic signs or were found dead without overt clinical signs. The onset of morbidity or death ranged from 4 to 8 HPAIV-dpc. Clinical signs varied from mild to severe and included lethargy, incoordination, paresis, walking in circles, and head circling and tremors. One wood duck in the naïve group showed a unilateral cloudy blue eye one day before it was found dead at 5 HPAIV-dpc.

Mortality varied greatly among groups, ranging from 0 to 100% (Table 1). All five wood ducks in the naïve group died, with a mean death time (MDT) of 5 days (range 4–6 days). Among the LPAI virus pre-exposure groups, the observed mortality was 80% (4/5) in the A/mallard/Netherlands/2/05 (H5N2) group (MDT of 6 days; range 6 days); 60% (3/5) in the A/blue-winged teal/LA/B228/86 (H1N1) group (MDT of 6 days; range 5–7 days); and 20% (1/5) in the A/mute swan/MI/451072-2/06 (H5N1) group (MDT of 8 days). Neither morbidity nor mortality was observed in the group pre-exposed to the A/mallard/MN/355779/00 (H5N2). Based on the number of birds that survived the Mongolia/05 virus challenge, the level of protection induced by the exposure to the LPAI viruses was statistically significant in the A/mallard/MN/355779/00 (H5N2) (*P* = 0.0079) and A/mute swan/MI/451072-2/06 (H5N1) (*P* = 0.0476) groups. The groups pre-exposed to the other two LPAI viruses (the A/blue-winged teal/LA/B228/86 [H1N1] and the A/mallard/Netherlands/2/05 [H5N2] groups) did not have a statistically significant level of protection against the Mongolia/05 virus (*P*>0.05).

The serological status of the wood ducks upon arrival had minimal if any effect on the outcome of Mongolia/05 virus infection, as demonstrated by similar mortality rates for birds within each group (Table 1). Statistical analyses revealed that the survival following Mongolia/05 virus challenge was not associated with the serological status upon arrival (χ^2^ = 0.987, *P*>0.05).

#### Viral Shedding

After challenge, the Mongolia/05 virus was isolated from every wood duck in each of the five groups. In general, viral shedding via oropharynx was greater than via cloaca, as demonstrated by a higher number of isolations from OP than cloacal swabs (Table 3). Viral shedding via oropharynx was consistent in all wood ducks from 1 to 4 HPAIV-dpc and was intermittent in individual birds up to 10 HPAIV-dpc. Cloacal shedding was less consistent that oropharyngeal shedding. Positive cloacal swabs were detected every day after Mongolia/05 virus challenge, and the highest number of virus isolation from cloacal swabs was at 4 HPAIV-dpc. Furthermore, cloacal shedding was detected more frequently in birds that succumbed to Mongolia/05 virus infection than in birds that survived.

**Table 3:** Clinical outcome and virus isolation data of wood ducks (*Aix sponsa*) challenged^a^ with the highly pathogenic avian influenza virus A/whooper swan/Mongolia/244/05 (H5N1).

Clinical Outcome^b^	Virus isolation, HPAIV-dpc^c^^,^ d													
	1		2		4		5^e^		6^e^		7		10	
	OP	CLO	OP	CLO	OP	CLO	OP	CLO	OP	CLO	OP	CLO	OP	CLO
Succumbed	12/13	1/13	13/13	4/13	13/13	11/13	4/12	4/12	4/8	4/8	0/2	0/2	NA	NA
Survived	10/12	0/12	10/12	2/12	9/12	1/12	*	*	*	*	1/12	0/12	1/12	3/12

a Birds were challenged via choanal cleft with a dose of 10^4^EID_50_ of A/whooper swan/Mongolia/244/05 (H5N1), 21 days after experimental pre-exposure to different subtypes of LPAI viruses.

b Five out of 13 birds that succumbed and six out of 12 birds that survived had pre-existing antibodies against avian influenza.

c Abbreviation: HPAIV-dpc = days post H5N1 HPAI challenge; OP = oropharyngeal swab; CLO = cloacal swab; NA = non applicable; * = not tested.

d no. of birds that shed the virus/total.

e Only birds that died were tested. Therefore, 4/12 birds were tested at 5 HPAIV-dpc, and 6/8 were tested at 6 HPAIV-dpc.

#### Necropsy Findings

Gross lesions were not detected in any of the wood ducks that survived the Mongolia/05 virus challenge and were necropsied at 10 HPAIV-dpc. All the wood ducks that succumbed to the Mongolia/05 virus infection had one or more of the following gross lesions: multifocal to coalescing areas (1- to 7-mm-diameter) of red mottling in the pancreas (10/13), congestion of meningeal vessels of the cerebrum and cerebellum (7/13), splenomegaly (5/13), multiple red foci in the adrenal glands (4/13), and a small tan spleen (2/13).

#### Histopathology and Immunohistochemistry

**Figure 1:**
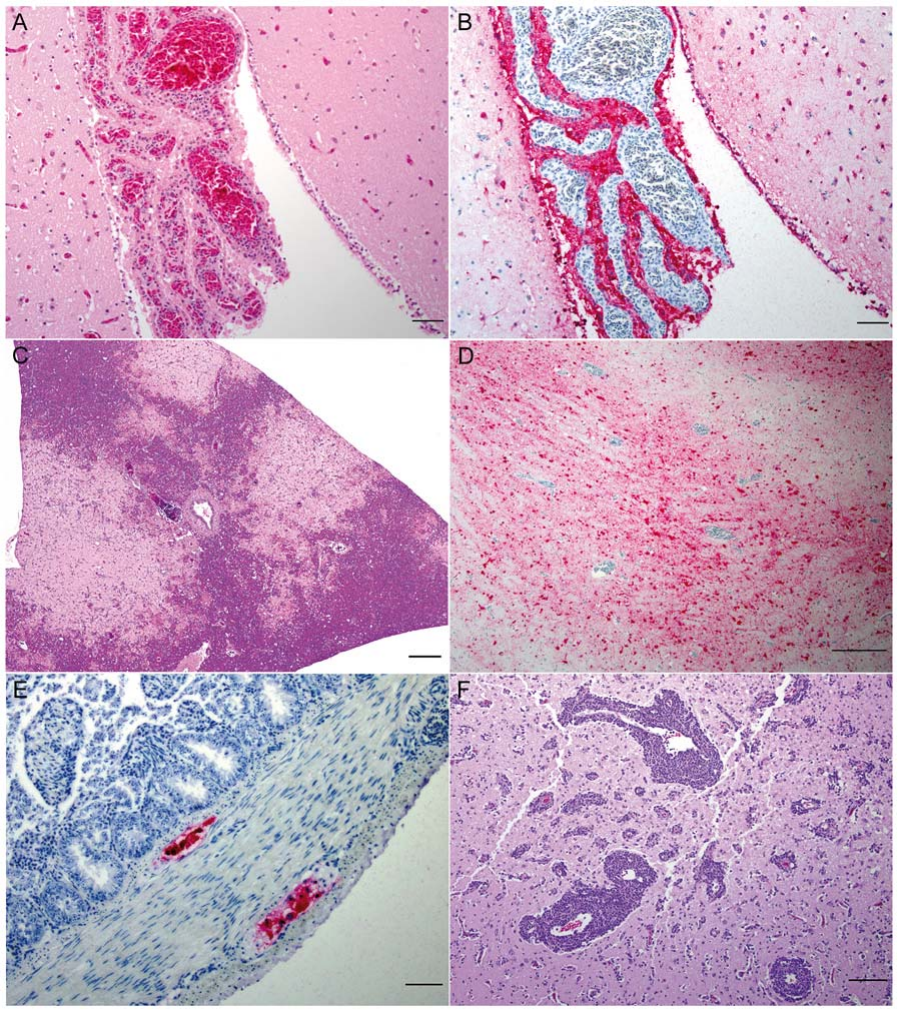
Histopathology and immunohistochemistry of different tissues from wood ducks (*Aix sponsa*) infected with A/whooper swan/Mongolia/244/05 (H5N1). (A) Brain, Choroid Plexus; wood duck that succumbed to Mongolia/05 virus infection. Severe choroiditis characterized by necrosis of epithelial cells of choroid plexus, vasculitis and heterophilic infiltrate. Hematoxylin-eosin staining. Bar 50µm. (B) Brain, Choroid Plexus; wood duck that succumbed to Mongolia/05 virus infection. Positive staining in epithelial cells of choroid plexus. Immunohistochemical staining: mouse-derived monoclonal antibody (P13C11), biotin-streptavidin alkaline-phosphatase detection method, Fast Red substrate-chromogen, hematoxylin counterstain. Bar 50µm. (C) Pancreas; wood duck that succumbed to Mongolia/05 virus infection. Severe multifocal to coalescing necrosis of acinar cells. Hematoxylin-eosin staining. Bar 200µm. (D) Brain, Cerebrum; wood duck that succumbed to Mongolia/05 virus infection. Intense positive immunohistochemical staining of neurons and glial cells. Immunohistochemical staining: mouse-derived monoclonal antibody (P13C11), biotin-streptavidin alkaline-phosphatase detection method, Fast Red substrate-chromogen, hematoxylin counterstain. Bar 200µm. (E) Small intestine; wood duck that succumbed to Mongolia/05 virus infection. Positive immunohistochemical staining in neurons of myenteric and submucosal plexus. Immunohistochemical staining: mouse-derived monoclonal antibody (P13C11), biotin-streptavidin alkaline-phosphatase detection method, Fast Red substrate-chromogen, hematoxylin counterstain. Bar 50µm. (F) Brain, Cerebrum; wood duck that survived the Mongolia/05 virus challenge. Severe nonsuppurative encephalitis characterized by marked perivascular cuffing. Hematoxylin-eosin staining. Bar 100µm.

The histopathologic lesions observed in wood ducks that succumbed to Mongolia/05 virus infection and those that survived are described in Table 4 and illustrated on Figure 1. Among the 13 wood ducks that succumbed to infection, the most consistent microscopic lesions were observed in the central nervous system (CNS), pancreas, liver, and adrenal gland. Lesions of the CNS were characterized by moderate to severe, multifocal to coalescing, nonsuppurative encephalitis and neuronal necrosis. Moderate to severe choroiditis (Figure 1A), meningitis, gliosis, vacuolar degeneration (associated or not with lymphoplasmacytic infiltrate), and perivascular cuffing were also observed in the CNS. Pancreatic lesions were characterized by severe multifocal to coalescing necrosis of acinar cells (Figure 1C). Hepatic changes included multifocal hepatocellular necrosis and periportal/centrilobular hepatitis. Severe multifocal to coalescing necrosis of corticotrophic and chromaffin cells was seen in adrenal glands. Other variably detected microscopic lesions included: neuritis and necrosis of myenteric plexus of small intestine, shortening and fusion of intestinal villi, orchitis, oophoritis, pulmonary edema, tracheitis, sinusitis, necrosis of follicles in cloacal bursa, and degeneration of sheathed capillaries, lymphoid necrosis, and subcapsular hemorrhage in spleen (Table 4).

**Table 4:** Histopathology lesions observed in wood ducks (*Aix sponsa*) that succumbed or survived after challenge with the highly pathogenic avian influenza virus A/whooper swan/Mongolia/244/05 (H5N1)^a^.

System/Tissue	Lesion	Succumbed (n = 13)	Survived (n = 12)
*Nervous*			
Cerebrum	Neuronal degeneration, gliosis, perivascular cuffing, vacuolar degeneration	12	2
	Choroid plexus, necrosis, inflammation	7	0
Cerebellum	Purkinje cell necrosis, gliosis, perivascular cuffing, status spongiosus, gitter cells	10	0
	Meninges, inflammation, necrosis	2	1
Brain Stem	Vacuolation, gliosis, perivascular cuffing, central chromatolysis	10	1
*Endocrine*			
Pancreas	Acinar cells, necrosis	8	0
Adrenal Glands	Corticotrophic and chromaffin cells, necrosis	3	0
*Digestive*			
Liver	Necrosis, multifocal	10	0
	Periportal/centrilobular hepatitis	5	10
Small Intestine	Myenteric plexus, neuronal necrosis	2	0
	Myenteric plexus, neuritis	0	2
	Villi, shortening and fusion	1	0
Large Intestine	Villi, shortening and fusion	1	0
*Reproductive*			
Testis	Seminiferous tubules, necrosis	1	0
Ovary	Immature follicle, necrosis, inflammation	1	0
*Respiratory*			
Lung	Edema	4	2
Trachea	Tracheitis	1	0
Nasal Sinus	Sinusitis	1	1
*Lymphoid*			
Cloacal Bursa	Necrosis, follicle	1	0
Spleen	Sheathed capillaries, degeneration	1	0
	Lymphoid necrosis	1	0
	Subcapsular hemorrhage	1	1
	White pulp, hyperplasia	0	4
	Amyloidosis	0	1

a Wood ducks were challenged via choanal cleft with a dose of 10^4^EID_50_ of A/whooper swan/Mongolia/244/05 (H5N1) 21 days after experimental pre-exposure to different subtypes of low pathogenic avian influenza viruses.

Positive IHC staining in association with histological lesions was consistently observed in nervous, digestive, and endocrine systems (Table 5, Figure 1) of the wood ducks that succumbed to Mongolia/05 virus infection, including: neurons and glial cells of cerebrum (Figure 1D), cerebellum, and brain stem, epithelial cells of choroid plexus (Figure 1B), neurons of myenteric and submucosal plexus of small and large intestines (Figure 1E), Kupffer cells and hepatocytes in the liver, acinar pancreatic cells, corticotrophic and chromaffin cells of adrenal gland, epithelial cells of trachea, epithelial cells of the seminiferous tubules, and granulosa cell layer of ovarian follicle. In wood ducks that succumbed to Mongolia/05 virus infection, positive immunohistochemical staining in the absence of microscopic lesions was routinely observed in cerebrum (4/13), cerebellum (3/13), brain stem (4/13), submucosal (8/13) and myenteric (5/13) plexus of small and large intestines, and sporadically in the meninges (1/13), salivary gland (1/13), cloacal bursa (1/13), testis (1/13), heart (2/13), nasal sinus (1/13), goblet cells of trachea (1/13), lung (1/13) and skin (2/13) (Table 5).

**Table 5:** Immunohistochemical analysis for nucleoprotein of avian influenza virus of wood ducks (*Aix sponsa*) that succumbed to infection (n = 13) with the highly pathogenic avian influenza virus A/whooper swan/Mongolia/244/05 (H5N1)^a^.

System/Tissue	Cell Type	No. of wood ducks	No. of positive cells^b^
*Nervous*			
Cerebrum	Neuron	8*	+++
	Epithelial cell of choroid plexus	8	+++
	Glial cells	6*	+++
	Ependymal cells	3	+++
	Endothelium of blood vessels	2	+
	Meninges	1**	++
Cerebellum	Purkinje cells	8	+++
	Neurons	5*	++
	Glial cells	7*	+++
Brain Stem	Epithelial cell of choroid plexus	6	+++
	Neurons	6*	+++
	Glial cells	6*	+++
	Endothelium	1	+
*Digestive*			
Salivary Gland	Epithelium	1**	+
Esophagus	Endothelium of submucosa	1	+
Intestines	Myenteric plexus	8*	++
	Submucosal plexus	9*	++
Liver	Kupffer cells	8	++
	Hepatocytes	1	++
*Endocrine*			
Pancreas	Acinar cells	6	+++
Adrenal Glands	Corticotrophic and chromaffin cells	4	+++
*Respiratory*			
Nasal Sinus	Epithelial cells	1**	+
	Perivascular staining	1**	+
Trachea	Epithelial cells	1	+
	Globet cells	1**	+
Lung	Epithelial cells	1**	+
	Secondary bronchus, submucosa	1**	+
*Integumentary*			
Skin	Epidermis, basal layer	2**	+
	Endothelial cells	1**	+
*Cardiovascular*			
Heart	Myocytes	2**	+
*Reproductive*			
Ovary	Follicle, granulosa cell layer	1	+
Testis	Seminiferous tubules	1	+
	Fibrovascular stroma	1**	+
*Lymphoid*			
Cloacal Bursa	Follicle, epithelial tuft	2	+
	Follicle, medulla	1**	+

a Wood ducks were challenged via choanal cleft with a dose of 10^4^EID_50_ of A/whooper swan/Mongolia/244/05 (H5N1) 21 days after experimental pre-exposure to different subtypes of low pathogenic avian influenza viruses.

b Numbers of immunohistochemically positive cells: + = few; ++ = moderate; +++ = numerous.

*Positive immunohistochemical staining associated or not with microscopic lesions.

**Positive immunohistochemical staining not associated with microscopic lesions.

Among the 12 wood ducks that survived Mongolia/05 virus challenge and were necropsied at 10 HPAIV-dpc, the microscopic lesions included periportal/centrilobular lymphoplasmacytic hepatitis (10/12), splenic white pulp hyperplasia (4/12), severe nonsuppurative encephalitis (associated or not with meningitis) and marked perivascular cuffing (3/12) (Figure 1F), neuritis of myenteric plexus of small intestine (2/12), pulmonary edema (2/12), and splenic amyloidosis (1/12) and subcapsular hemorrhage (1/12) (Table 4).

## Discussion

Wood ducks used in this study were acquired from a duck farm where several species of Anseriform and Galliform birds were raised outdoors and had direct contact with wild birds. Outdoor rearing of poultry is a major risk factor for exposure to AI virus [30], and could explain the 12 wood ducks that tested positive for influenza A group-specific antibodies prior to the start of the trial. Further testing revealed that all 25 wood ducks were seronegative on the HI test, indicating that none of the birds had pre-existing antibodies directed against the HA of the LPAI viruses used for pre-exposure or the Mongolia/05 challenge virus. Although unexpected, the presence of individual birds with pre-existing antibodies to influenza A nucleoprotein, which are non-neutralizing and non-protective [31], [32], did not preclude us from acquiring useful data from this study. As shown on Table 1, within each of the LPAI virus-exposure groups, there was minimal differences in mortality (more or less than one bird) between wood ducks that had antibodies prior to the study and those that did not. This was further confirmed through statistical analyses, which revealed that the survival following Mongolia/05 challenge was not influenced by the serological status prior to the study.

The influence of pre-exposure to a LPAI virus on the outcome of a HPAI challenge varied from total protection (0% morbidity/mortality) to no protection (100% mortality) (Table 1). All the wood ducks in the naïve group died, confirming the high susceptibility of this species to Mongolia/05 virus observed in previous studies [23], [24]. The North American A/mallard/MN/355779/00 (H5N2) and A/mute swan/MI/451072-2/06 (H5N1) viruses (both homosubtypic to Mongolia/05 challenge virus) induced a statistically significant level of protection, as evidenced by low mortality (0 and 20%, respectively) after H5N1HPAI virus challenge (Table 1). Alternatively, the Eurasian A/mallard/Netherlands/2/05 (H5N2) virus (also homosubtypic to Mongolia/05 challenge virus) did not replicate in the wood ducks and, consequently, provided no protective immunity. This was reflected by the high mortality (80%) in this group. The wood ducks pre-exposed to the A/blue-winged teal/LA/B228/86 (H1N1) virus (heterosubtypic to Mongolia/05 challenge virus) experienced moderate mortality (60%) relative to the other exposure groups; the level of protection observed in this group was not statistically significant. These observations suggest that humoral immunity against homosubtypic LPAI viruses play an important role in providing protection against H5N1 HPAI virus infection, as observed in previous studies [32], [33], [34]. These results also indicate that the development of a protective immune response is dependent on detectable replication of the LPAI virus.

An adequate humoral immune response to HA seems to be fundamental for the protection against challenge with H5N1 HPAI virus in multiple avian species [33]. In the current study, three wood ducks that succumbed to H5N1 HPAI virus infection (two birds in the A/blue-winged teal/LA/B228/86 [H1N1] group and one bird in the A/mute swan/MI/451072-2/06 [H5N1] group) had a negative HI titer against the homologous HA at 21 LPAIV-dpe when they were subsequently challenged with the Mongolia/05 virus (Table 2). Moreover, a higher HI titer against the homologous LPAI isolate was observed among surviving birds, particularly in wood ducks in the A/mute swan/MI/451072-2/06 (H5N1) and A/mallard/MN/355779/00 (H5N2) groups (Table 2). This increase in titer is not understood, but could either be an effect of the H5N1 HPAI virus challenge or related to the timing of the original immune response (31 LPAIV-dpe). It is currently not understood whether cell mediated immunity (CMI) is involved in the protective immune response against H5N1 HPAI virus. However, the wood duck in the A/mallard/Netherlands/2/05 (H5N2) group that survived the Mongolia/05 virus challenge did not develop HI detectable antibody against the homologous H5 used for pre-exposure (data not shown), suggesting that humoral immunity is not the only factor influencing the outcome of infection. Further investigations are needed to clarify the roles of humoral immunity against HA and NA and CMI in protection of birds against H5N1 HPAI virus challenge.

The Mongolia/05 virus replicated systemically in wood ducks and had a strong tropism for the CNS, pancreas, and adrenal gland. Failure and/or dysfunction of one or multiple of these organs were likely the cause of death. The cloudy eye observed in one wood duck in the naïve group has been frequently reported as a clinical sign of H5N1 HPAI virus infection in birds [17], [23], [35], [36], [37], [38], [39]. Although histopathologic examination was not performed on the eye of the wood duck in this study, the cloudiness may be caused by the deposition of fibrin or accumulation of white blood cells in the anterior chamber or corneal edema, both of which can be features of uveitis.

Consistent with other experimental studies, oropharyngeal shedding in all five wood duck groups was more pronounced than cloacal shedding (Table 3) [17], [23], [24], [35], [39]. In this study, viral shedding was not completely suppressed in any of the groups of wood ducks pre-exposed to a LPAI virus, contrary to what was observed in a previous experimental investigation with mallards [17]. Although wood ducks that succumbed to infection died between 4 and 8 HPAIV-dpc, the cloacal viral shedding observed in these birds was more prominent (based on number of birds shedding) than in birds that survived the HPAI virus challenge (Table 3). Presumably, this is the result of widespread viral replication in more severely affected wood ducks, which is supported by the presence of viral antigen in adrenal glands, pancreas, liver, myenteric and submucosal plexus of the intestines, and reproductive organs (ovaries and testicle) (Tables 4 and 5, Figure 1). Viral antigen in the pancreas and reproductive tissues is particularly relevant, as viral replication in these organs could contribute to fecal shedding. Viral titrations of cloacal and OP swabs were not performed in this study; therefore, the influence that pre-exposure to a LPAI virus has on the concentration of Mongolia/05 virus shedding in wood ducks is not known. A recent study observed that a previous exposure to a LPAI virus can reduce the concentration and duration of viral shedding via oropharynx and prevent cloacal shedding in mallards infected with H5N1 HPAI virus [17].

In summary, this study demonstrates that previous exposure to homosubtypic LPAI viruses in wood ducks may: prevent disease but not infection or viral shedding of H5N1 HPAI virus; decrease mortality associated with H5N1 HPAI virus infection; and increase MDT, thus prolonging the duration of H5N1 HPAI viral shedding. Furthermore, the degree of protection against the H5N1 HPAI virus varies in response to the LPAI virus subtype that the bird was previously infected with. The mechanisms responsible for this protection, as well as the extent (between different subtypes) and duration of this protective immunity still need to be elucidated in order to understand the epidemiology of AI viruses in waterfowl reservoirs and to better define the potential risks of unique AI viruses, such as H5N1 HPAI virus, to infect, move with, or become established in wild bird populations.
